# College and Hospital Notes

**Published:** 1909-04

**Authors:** 


### COLLEGE AND HOSPITAL NOTES

---

The directors of the German Hospital, it is stated, have decided to build a nurses' home on the lot adjacent to the hospital on Jefferson street, Buffalo. All the money needed, it is said, has been subscribed. The home will cost about $12,000.

The officers of the hospital for the current year are: honorary president, William Simon; president, William F. Kasting; vice-president, Dr. C. H. W. Auel; secretary, Harry J. Knepper; treasurer, Henry vom Berge; auditing committee, Ottomar Reinecke and Jacob J. Lang; membership committee, Henry C. Zeller and Joseph G. Schaff.

The Buffalo General Hospital Alumni Association held its annual dinner February 19, 1909, at the Hotel Statler. Dr. Peter W. Van Peyma was toastmaster and the speakers were Drs. Charles G. Stockton, Carlton C. Frederick, Marcell Hartwig, Renwick R. Ross and John H. Pryor. Dr. Roswell Park read a paper on "The Next Quarter Century in Sugery." Between the dinner and the speeches an entertaining minstrel show was enjoyed by the members. Seventy-nine covers were laid.

---

The Howe Eye Hospital recently filed incorporation papers in the County Clerk's office. The directors are Dr. Lucien Howe, Elizabeth M. Howe, Sallie B. Howe, William Delancey Howe, James N. Adam, Charles P. Norton and Roswell Park.

---

The Waldorf-Astoria was the first New York hotel to recognise the importance of its own hospital, and every one said when the emergency ward was opened in the autumn: "No one but George Boldt would ever have thought necessary or carried out such a project in a hotel." For a long time a prominent firm of chemists has conducted a branch shop in the basement of the Waldorf, which has been kept open all night long. One of the basement rooms was lined with white tiling, and there was installed therein an operating table, rubber tired roller couch, cases of surgical instruments and a complete sterilising outfit. The resident house physician at the Waldorf is in charge of the room, with his brother as his assistant and a professional nurse in attendance.

---

Bright, airy, spotlessly clean, modern in every detail from the receiving room to the solarium on the roof, with hardly a square inch in it that the hose cannot be turned upon, the new Jewish Maternity Hospital at Nos. 270 and 272 East Broadway, was dedicated at 2 p. m., Sunday, January 17, 1909. The building will cost $90,000, and the $30,000 raised so far came in voluntary contributions, in sums ranging from 25 cents to $500. On voluntary contributions it will have to subsist, for there is no fund, no endowment, and every one of the sixty beds are free. Though built by Jews, it will be non-sectarian; any woman, Jew or Gentile, Catholic or Protestant, who needs its care will be received.

The room in which the dedication exercises was held, on the first floor, is the one the physicians and board of directors are proudest of. This room is to be a nursery where the children of the patients will be taken care of by a matron and her helpers.

"Most of these poor mothers have little children," said Dr. A. M. Hilkowich, secretary of the board of directors. "There is no one to leave them with at home, and a woman will refuse to go to the hospital—she would rather be confined in her unsanitary tenement home, often consisting of a kitchen and one little dark room, where there is no gas and one must attend her by the light of a candle or a smoky lamp—she would rather stay there than leave her children. That is why we felt the nursery to be an important part of the hospital. The mother can be content, knowing her children are well cared for."

All the floors of the hospital are of hard wood covered with shellac. The walls are painted a pale yellow, the metal work pure white. The lights in the wards are arranged so that there will be no glare—just a soft, diffused glow.

There are a few private rooms. The charge will be low for these, and if a patient needs to be alone and has no money, the hospital will try to find the money. There will be fifteen nurses, and an obstetrical training school will be maintained in connection with the hospital.

The attending physicians are Dr. J. Heller, Dr. A. J. Rongg, Dr. N. Ratnoff and Dr. A. M. Hilkowich. The consulting physicians are Dr. Ralph Waldo and Dr. S. W. Bandler. The Rev. Philip Joches is president of the hospital. The organisation got its charter two years ago, and since then has done outdoor relief work, taking care of about eight hundred women. It was in the course of that work that the sore need of a hospital for maternity cases in that part of the city was seen.

At the dedicatory exercises Mrs. William Einstein, on behalf of the women of New York, formally received the key of the hospital from the board of directors.

---

McGill University Celebrates.—Fifty graduates of McGill University, Montreal, drank a toast to "The President; the King," in the college-room of the Hotel Astor, Thursday night. March 25, 1909, and with a shout of "they are jolly good fellows," started in to prove that New York and McGill are not so far apart after all. They succeeded all the better because they had Dr. William Peterson, president of the university, and Dr. Frank D. Adams, dean of the faculty of sciences, to give the graduates a bit of Canadian atmosphere.

Dr. Peterson, in his speech about the university, told the alumni one thing that brought forth cheers. The Carnegie Foundation has appointed a committee to go around the country and assess the value of the medical educations taught in the various colleges. This is with the purpose of improving the curricula generally. that better physicians may be graduated.

Dr. Peterson told the McGill men that the committee called on him a short time ago. He called on Mr. Pritchett, the head of the Carnegie Foundation, today, and was told that McGill medical school would go way to the top of the list; that only

Harvard and Johns Hopkins approached it in excellence. Another thing that Dr. Peterson called attention to was the great work McGill is doing in bringing together the various races in Quebec Province, molding them into a unit, "without which Canada will never be a great nation."

Dr. Peterson said that without wishing to talk finances at all, he was going to smite the people of Toronto for the way in which they treated McGill. If McGill were a corporation, he said, the directors would have to close up shop. He intimated that Lord Strathcona and Sir William McDonald, although he did not mention them by name, had come to the rescue of McGill and prevented the threatened shutdown. Montreal was singularly apathetic and although the citizens gave some financial assistance, it was nothing compared to what they could do if they would.

The other speakers were Dean Adams, C. W. Miller of Queens College and H. F. Ballantyr of Toronto.

